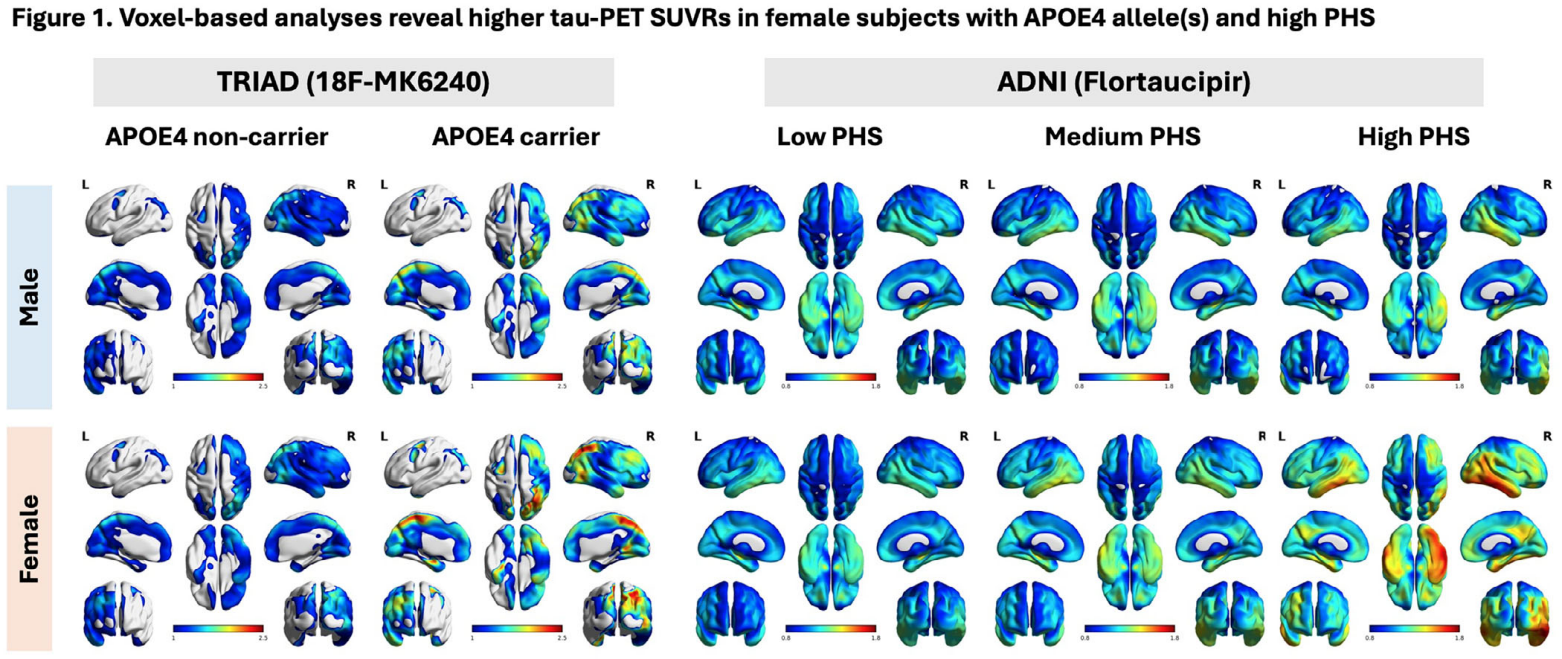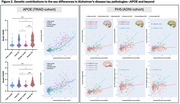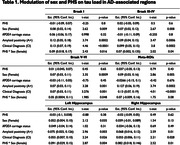# Genetic contribution to the sex differences of tau pathologies in Alzheimer’s disease –APOE and beyond

**DOI:** 10.1002/alz.092470

**Published:** 2025-01-09

**Authors:** Yi‐Ting Wang, Joseph Therriault, Nesrine Rahmouni, Arthur C. Macedo, Stijn Servaes, Jaime Fernandez Arias, Sulantha Mathotaarachchi, Jenna Stevenson, Serge Gauthier, Pedro Rosa‐Neto, for the Alzheimer’s Disease Neuroimaging Initiative

**Affiliations:** ^1^ McGill University, Montreal, QC Canada; ^2^ McGill University Research Centre for Studies in Aging, Montreal, QC Canada; ^3^ Translational Neuroimaging Laboratory, The McGill University Research Centre for Studies in Aging, Montréal, QC Canada; ^4^ Translational Neuroimaging Laboratory, The McGill University Research Centre for Studies in Aging, Montreal, QC Canada; ^5^ McConnell Brain Imaging Centre, Montreal Neurological Institute, McGill University, Montreal, QC Canada; ^6^ Department of Neurology and Neurosurgery, McGill University, Montréal, QC Canada; ^7^ Department of Psychiatry, McGill University, Montreal, QC Canada

## Abstract

**Background:**

Alzheimer's disease (AD) disproportionately impacts females, who exhibit a higher tau load compared to males. Existing literature suggests that biological sex may alter the impact of APOE on tau pathology in AD. Nevertheless, the genetic factors contributing to sex differences in AD tau pathology, beyond the influence of APOE, have been minimally investigated.

**Methods:**

This study included 2 well‐characterized AD cohorts: the Translational Biomarkers in Aging and Dementia (TRIAD, n=359) cohort and the ADNI cohort (n=283). All participants underwent APOE genotyping and PET imaging with radioligands targeting Aβ plaques and tau tangles respectively. Polygenic hazard score (PHS) was computed using 31 AD‐associated SNPs identified from a stepwise Cox proportional hazards model for each ADNI participant. Low and high PHS is defined as low: −1 SD, ∼16 percentile; and high: +1 SD, ∼84 percentile.

**Results:**

Sex‐disaggregated voxel‐based analyses revealed higher tau‐PET SUVRs in female subjects with APOE4 allele(s) and high PHS (Figure 1). ANOVA tests also showed a significant difference in tau load in Braak I‐II ROIs between female E4 carriers and both male E4 carriers (P<0.05) and non‐carriers (P<0.0001). To illustrate the relationship between Aβ and tau, modulating by APOE4carriage status, non‐linear Locally Weighted Scatterplot Smoothing (LOWESS) trendlines were applied, demonstrating an initial rise in tau among female E4 carriers, succeeded by male E4 carriers (Figure 2). Subsequently, linear Ordinary Least Squares (OLS) regression analyses were conducted to explore the influence of PHS on the Aβ‐tau relationship. Notably, female individuals in the high PHS group exhibited the highest tau load under a comparable Aβ load compared to other groups (Figure 2). In line with this, regression analyses unveiled a modulating effect of PHS and sex on tau load in AD‐associated regions, and this effect remained statistically significant after adjusting for APOE4 carriage status (Table 1).

**Conclusions:**

In the trajectory of Alzheimer's disease, early‐stage tau deposition was more pronounced in female individuals with the APOE4 allele. Our findings suggest exploring the polygenic architecture aids in identifying female individuals at the highest risk of developing tau pathology. This may be valuable for stratifying AD risk and as an enrichment strategy in clinical trials.